# Towards Therapeutic Delivery of Extracellular Vesicles: Strategies for* In Vivo* Tracking and Biodistribution Analysis

**DOI:** 10.1155/2016/5029619

**Published:** 2016-11-23

**Authors:** Giuliana Di Rocco, Silvia Baldari, Gabriele Toietta

**Affiliations:** Department of Research, Advanced Diagnostic, and Technological Innovation, Translational Research Area, Regina Elena National Cancer Institute, Via E. Chianesi 53, 00144 Rome, Italy

## Abstract

Extracellular vesicles (EVs), such as microvesicles and exosomes, are membranous structures containing bioactive material released by several cells types, including mesenchymal stem/stromal cells (MSCs). Increasing lines of evidences point to EVs as paracrine mediators of the beneficial effects on tissue remodeling associated with cell therapy. Administration of MSCs-derived EVs has therefore the potential to open new and safer therapeutic avenues, alternative to cell-based approaches, for degenerative diseases. However, an enhanced knowledge about* in vivo* EVs trafficking upon delivery is required before effective clinical translation. Only a few studies have focused on the biodistribution analysis of exogenously administered MSCs-derived EVs. Nevertheless, current strategies for* in vivo* tracking in animal models have provided valuable insights on the biodistribution upon systemic delivery of EVs isolated from several cellular sources, indicating in liver, spleen, and lungs the preferential target organs. Different strategies for targeting EVs to specific tissues to enhance their therapeutic efficacy and reduce possible off-target effects have been investigated. Here, in the context of a possible clinical application of MSC-derived EVs for tissue regeneration, we review the existing strategies for* in vivo* tracking and targeting of EVs isolated from different cellular sources and the studies elucidating the biodistribution of exogenously administered EVs.

## 1. Introduction

Mesenchymal stem cells (MSCs) are a heterogeneous subpopulation of cells with self-renewal and multilineage differentiation abilities, present in the stromal fraction of many adult tissues [1]. MSCs are expanded* in vitro* upon selection by adherence to plastic surfaces [2]. In order to define common standards, the International Society for Cellular Therapy set minimal criteria for defining MSCs and suggested the use of the term “mesenchymal stromal cells” (maintaining the acronym MSCs) for the designation of the plastic-adherent cells previously defined as “mesenchymal stem cells” [3, 4]. Indeed, the definition of MSCs is continuously evolving, taking into account more recent understanding in MSCs biology [5]. Several animal and human studies provided the proof-of-concept for the use of MSCs transplantation for the treatment of diseases associated with tissue degeneration [6]. It was originally assumed that MSCs exert their therapeutic effect on tissue regeneration mainly by differentiating into specialized cells able to repopulate the injured tissue. Increasing evidences have demonstrated that the fraction of administered cells that actually survives upon transplantation, engrafts, differentiates, and provides functional support for tissue regeneration is minimal [7]. Moreover, some beneficial effects have been observed upon administration of factors secreted by MSCs [8]. These observations suggest that the prevalent mechanism by which MSCs exert their contribution to tissue regeneration is mostly associated with their paracrine activity [9–13]. Accordingly, MSCs secretome can be viewed as a remarkable tool for regenerative medicine, which poses reduced safety concerns and easier technological processes for production and storage compared to cell-based therapeutics [14, 15]. Indeed MSCs secrete a wide variety of factors with proangiogenic, anti-inflammatory, antiapoptotic, and immunomodulatory properties [7]. Moreover, molecules secreted by MSCs include modulators of cellular growth, replication, differentiation, and adherence [16]. Several studies are currently focused on uncovering the nature of MSCs secretome [17, 18], which consists of both soluble factors such as cytokines, chemokines, growth factors, and other proteins, lipids, and nucleic acids, released within extracellular vesicles. How MSCs secretome exerts its beneficial effects on tissue regeneration has not been fully elucidated yet [19]. Due to the heterogeneous nature of MSCs also the mechanism of action of MSCs secretome can be multifaceted [10]. Paracrine factors may promote homing and activation of endogenous stem/progenitor cells, stimulate extracellular matrix remodeling, suppress apoptosis, limit local inflammation, reduce fibrosis, mediate chemoattraction, and support angiogenesis [7, 16]. A better understanding of the molecular and biochemical pathways targeted by MSCs paracrine effectors is crucial for clinical translation of secretome-based therapy approaches [14].

## 2. Cell-Derived Vesicles

Extracellular vesicles (EVs) are small membrane-enclosed particles derived from a variety of cell types including endothelial cells, dendritic, B and T cells, embryonic and mesenchymal stromal cells, neurons, oligodendrocytes, Schwann cells, intestinal epithelial cells, and platelets [20, 21]. EVs can be found in body fluids such as blood, urine, milk, saliva, amniotic, cerebrospinal, synovial and bronchial lavage fluids, and malignant effusions [22].

The definition “extracellular vesicles” encompasses vesicles with different origin, size, membrane composition, and content such as exosomes, microvesicles, microparticles, ectosomes, oncosomes, prostasomes, and apoptotic bodies [20, 23]. Distinction between different EV subgroups is difficult, due to the minimal physical and morphological differences, to the lack of specific markers, and to the fact that the same cellular source may dynamically produce different class of EVs in response to different conditions [21]. Currently there is no single method allowing for accurate characterization and discrimination of the different EVs classes [24, 25]. In fact, due to their small size, EVs cannot be resolved by light microscopy, neither be analyzed by conventional flow cytometry, but alternative, more cumbersome methods (recently reviewed by Rupert et al. [26]) should be used. In addition, differential centrifugation, which is considered the gold standard method used to isolate EVs, allows for enrichment, rather than purification of the various EVs populations [24, 27–29]. Other methods of isolation may result in different yields, making the direct comparison between various studies difficult [20].

In order to provide criteria for standardization of the nomenclature and the procedures for isolation and characterization of different EV subgroups, the International Society for Extracellular Vesicles has published in a position paper in 2004 [30]. International consensus has been achieved on the following classification: based on their biogenesis EVs can be divided into three main subclasses: (i) microvesicles, which originate directly from the shedding of the plasma membrane; (ii) apoptotic bodies which are generated upon activation of apoptotic pathways; and (iii) exosomes which are secreted by reverse budding of multivesicular bodies. Interestingly, the existence of distinct subpopulations of exosomes has been recently described [31], but further research is required to fully define exosome subclasses. Detailed description of biogenesis, secretion, and intercellular interaction of EVs has been extensively reviewed elsewhere [32]. The different classes of EVs have also been defined by their approximate diameter size: apoptotic bodies (1–5 *μ*m), microvesicles (100–1000 nm), and exosomes (40–100 nm) [33], but this classification is considered less accurate due to the intrinsic difficulties in performing precise measurements [34]. Further elucidation and a historical perspective on what can be defined as “exosome” were recently provided by Edgar [35]. Nonetheless, difficulties in accurately isolating and characterizing exosomes and other extracellular vesicles prompted some authors to apply the generic term “extracellular vesicles” to collectively denote vesicles obtained from biological samples or cell culture supernatants [34]. Accordingly, in this work we used the notation “extracellular vesicles” (EVs) for all secreted vesicles, although some of the cited articles specifically refer to “exosomes” or “microvesicles.”

**Figure 1:**
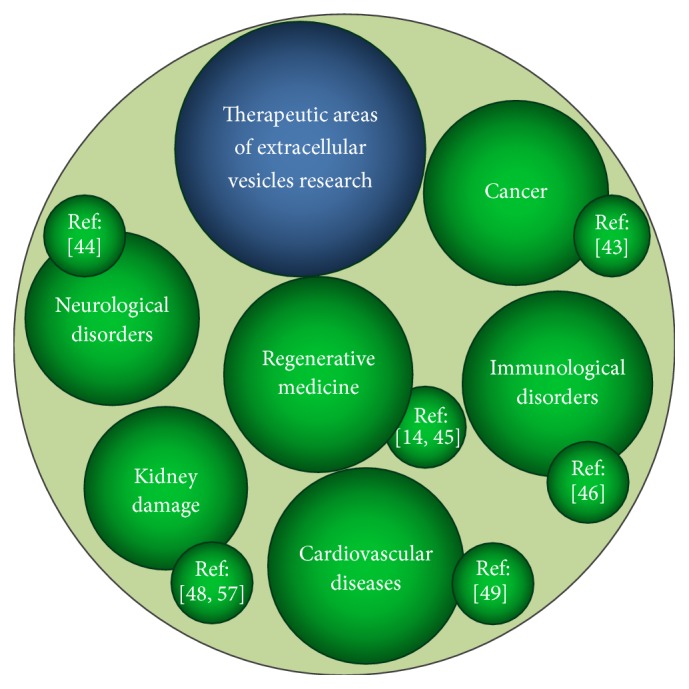
Main areas of potential therapeutic use of mesenchymal stem/stromal cells-derived extracellular vesicles.

Originally, EVs were considered as cellular debris without significant biological function. Actually, accumulating evidences indicate that EVs play a key role in intracellular signaling, exerting specific effects on homeostasis maintenance, modulation of the immune response, inflammation, cancer progression, angiogenesis, and coagulation, in both physiological and pathological conditions [21, 22, 33]. Detection of EVs in biological fluids can be used as diagnostic, prognostic, and treatment monitoring biomarker [36]. EVs lipid bilayer membrane includes transmembrane proteins and encloses soluble proteins and nucleic acids derived from the cell of origin [37]. EVs are able to shuttle protein, lipids, carbohydrates, messenger RNAs, long noncoding RNAs, microRNAs, mitochondrial DNA, and chromosomal DNA into target cells [38, 39]. Transferring distinct biomolecules, EVs mediate different signals between cells and organs, promoting tolerance to external stress stimuli such as inflammation, hypoxia, and oxidative and shear stress [40]. For this reason, EVs have increasingly been under investigation as novel modulators for different therapeutic purposes, including anticancer strategies, vaccination, targeted drug delivery, immunomodulation, and tissue regeneration [22, 41, 42]. Therefore, several possible applications for EVs-mediated therapy have been proposed (Figure 1) [14, 43–49].

Albeit several regulatory and technical issues in achieving highly purified and extensively characterized EVs preparations suitable for use in humans need to be solved [37, 50], several clinical trials have been conducted. Ohno et al. [41] recently reviewed the results of the phase I clinical trials of EVs-based therapies. Overall, no serious acute events have been associated with EVs administration [24, 50]. These preliminary trials have generated great expectation for ongoing and future clinical trials using EVs isolated from MSCs for tissue regenerative purposes [51].

## 3. Mesenchymal Stem/Stromal Cells-Derived Extracellular Vesicles and Tissue Regeneration

Studies using MSCs are the most prevalent among the cell-based therapies being tested for tissue regeneration, the reason being that (i) MSCs can be isolated from different, easily accessible, adult tissue sources, including bone marrow [83] and adipose tissue [84]; (ii) they can be cultured* in vitro*; and (iii) they can be induced into osteogenic, chondrogenic, adipogenic, endothelial, cardiovascular, neurogenic, and hepatic differentiation. The ability of MSCs to secrete a variety of growth factors, cytokines, and chemokines potentially involved in tissue repair and remodeling is well-established, being described for the first time 20 years ago [85]. Nonetheless, elucidating the factors that contribute to the regenerative ability of MSCs remains one of the most relevant but unresolved issues in the field. Therefore, in recent years, the attempts for identifying MSCs-secreted mediators with therapeutic potential have shifted from growth factor and cytokines to extracellular vesicles [86]. The existence of EVs is well-documented since the description of extracellular vesicles, termed “exosomes,” was first published nearly 30 years ago [87]. However, more recent studies demonstrating the ability of MSC-secreted EVs in providing protection against acute kidney damage [88], hepatic fibrosis [89], and myocardial [90] injury have sprouted a new interest on possible exploitation of EVs as therapeutic vehicles [22]. EVs may play a role in local tissue repair affecting progenitor cell proliferation, recruitment, and differentiation; promoting extracellular matrix remodeling and angiogenesis; overpowering apoptosis and immunological responses [45, 91].

EVs play a pivotal role in stem cell plasticity and tissue regeneration, possibly contributing to the paracrine action observed upon MSCs cell transplant [92, 93]. Purification of EVs released from cultured MSCs and their delivery to damaged tissues may represent a novel “acellular” therapeutic approach in the arena of regenerative medicine [14, 51]. This strategy can be considered as an alternative to cell-based therapeutic approaches [94], albeit MSCs are still necessary as EVs source. MSCs are a proficient source of EVs, including exosomes, which therefore can be obtained in a clinical relevant scale with procedures compliant with good manufacturing process standards [95]. Also immortalized MSCs produce considerable amounts of exosomes and microvesicles, making possible the generation of stable cell lines for consistent production of EVs [96]. In addition, genetic manipulation of producer cells might be used in order to increase production or to generate “tailored” EVs [79, 97]. EVs can be isolated from cells obtained from each patient, posing no question of immunocompatibility and allowing for repeated administration. Moreover, EVs-mediated delivery of biological material has improved safety profile compared to the current methods of delivery based on liposome and viral based vehicles. Favorably, EVs are fairly stable under different storage conditions [98], making them easier to store and deliver compared with living cells used in cell-based therapies.

## 4. Investigating Extracellular Vesicles Biodistribution by Molecular Imaging

The use of MSC-derived EVs for regenerative therapy requires production and isolation of a suitable quantity of clinical grade EVs from cultured MSCs [94]. For safe and successful clinical applications of EVs-based therapies for tissue regeneration, a better understanding of EVs biodistribution upon administration is needed [50]. A large amount of preclinical studies on the therapeutic potential of MSCs-derived EVs (recently reviewed by Akyurekli et al. [99]) has been performed. Nonetheless, current knowledge of the biodistribution of EVs upon administration in animal models is limited. To our knowledge, only one work evaluated the biodistribution of human bone marrow-derived MSC in murine models [58]. In the current section, we review the methods for EVs labeling and the biodistribution studies, including those performed by administration of EVs collected from cellular sources other than MSCs.

### 4.1. Methods for Extracellular Vesicles Labeling

Several strategies have been employed for* in vivo* tracking to determine EVs biodistribution upon systemic delivery in different animal models (Table 1) [100]. The ideal method should be specific, have a high signal-to-noise ratio, and mirror EVs half-life. Unfortunately, the methods currently used present some limitations. One approach consists, for instance, in loading EVs with superparamagnetic iron oxide nanoparticles for high resolution and sensitive magnetic resonance analysis [56]. Radioisotope labeling of EVs using clinically validated radio tracers and nuclear imaging have also been used for tracing EVs in murine experimental models [54, 55]. These techniques provide for accurate detection also in deep organs, but require instruments not available in many research departments.

Alternatively, EVs can be conveniently labeled with fluorescent dyes; both dyes selective for DNA and RNA contained in the EVs [101] and lipophilic dyes for labeling membrane components have been used [102–104]. Near-infrared (NIR) dyes are ideal for* in vivo* applications due to their high signal/noise ratio, the minimal autofluorescence of biological tissue in the 700–900 nm spectral range, and the strong tissue penetration of the near-IR light. In particular, the carbocyanine DiOC18(7) (DiR) is a lipophilic dye weakly fluorescent in water, but particularly fluorescent and photostable when incorporated into lipid-membranes. Lipophilic NIR dyes have been quite extensively employed for labeling of EVs isolated from different sources and administered into different animal models (Table 2). The major limitation, however, is that lipophilic dyes labeling promotes EVs aggregation and may give rise to artifacts, especially* in vivo* [59]. Moreover, extensive washing steps, needed to reduce the presence of dye residues which might result in nonspecific signals, can cause significant EVs loss. Nonetheless, valuable information on localization of EVs administered by different routes has been acquired using this labeling strategy followed by* in vivo* fluorescence optical imaging. Little is known about EVs' half-life after systemic administration. Recent evidences, obtained following miR loaded EVs expression, suggest that, in the blood, EVs are detectable as early as 5 min after intravenous administration, decrease by ~50% in 30 minutes, and become undetectable after 4 hours [105]. On the other hand, lipophilic dye staining is quite stable, with an* in vivo* half-life estimated in several days. Therefore, in long-term studies the extended half-life of the lipophilic dye may result in the maintenance of the fluorescent signal for longer than the EVs persistence itself [58]. To circumvent this problem, we have developed a method for EVs labeling without the use of fluorescent dye. The strategy is based on the genetic modification of the EVs-producing cells with a lentiviral vector derived from the X-Pack plasmid (System Biosciences, Palo Alto, CA) in which the coding sequence of the fluorescent protein TurboFP635 (Katushka red) (Evrogen, Moscow, Russia) has been cloned in frame with a specific peptide sequence that targets the protein into the EVs (Baldari et al., unpublished data). The choice of the Katushka far red fluorescent protein makes this labeling strategy suitable for* in vivo* imaging studies, due to reduced auto fluorescence in biological tissue in the near-infrared-shifted emission spectra [106]. This labeling method allows for the generation of a producer cell line which continuously secretes EVs containing the reporter protein of choice for downstream applications. Another method of EVs labeling has been recently used by Lai et al. directing the expression of fluorescent markers into the exosomal membrane by the generation of enhanced green (EGFP) and tandem dimer tomato (tdTomato) fluorescent proteins containing specific palmitoylation signals, which promote the membrane association of the proteins [67]. Albeit the range of fluorescent probes suitable for EVs labeling is continuously expanding, one of the major limitations for* in vivo* tracking studies is associated with the fact that fluorescent markers should have an emission peak not coinciding with the fluorescence emission of biological tissues, in order to overcome the autofluorescence background. Moreover, the use of fluorescent conjugated markers directed against specific proteins, such as CD63-GFP, may restrict labeling to specific subpopulations of EVs [58]. On the other side, fluorescent dyes for EV lipid labeling, such as the most commonly used PKH67 [60], are not EV-specific [67]. Consequently, they not only label EVs but also can be retained in association with other lipid entities for long periods, eventually forming aggregates or micelles, thus inducing false positive results [59]. In contrast, the palmitoylated fluorescent EV reporters, like PalmGFP and PalmtdTomato, have increased specificity compared with CD63-GFP and to PKH67 dye, allowing for labeling and semiquantification of multiple and different sized EV types, irrespective of their biogenesis, time-lapse live-cell imaging of EV release and uptake, and EV exchange between different cell populations [67].

Compared to fluorescent-based imaging, bioluminescent optical imaging (BLI), which uses luciferase enzymes as imaging reporters, has an extremely low signal-to-noise ratio, since the autoluminescence in mammalian tissue is negligible. In particular, the adapted bioluminescence reporters, such as Gaussia luciferase, being over 1,000-fold brighter than firefly luciferase, are useful tools to study temporal properties of minute biological processes because of their sensitivity, low background and independence from an excitation source to emit light. Therefore, BLI has been extensively evaluated in the development of cell-based therapies to determine cellular distribution, survival, proliferation, and differentiation after transplantation [107, 108]. BLI has also been described for the analysis of EVs associated with a luciferase enzyme. In particular, Takahashi et al. generated a fusion protein named gLuc-lactadherin consisting of the Gaussia luciferase (gLuc) enzyme combined with portions of the membrane protein lactadherin which are required for the protein translocation into the exosomal compartment and for retention on the exosomal membrane [62]. Cellular expression of gLuc-lactadherin results in production of EVs containing Gaussia luciferase on their membrane, which can be therefore detected by BLI. Using a similar approach Lai et al. generated a fusion between a membrane-bound variant of the gLuc reporter and a biotin acceptor peptide [64]. These reporters were instrumental for performing* in vivo* biodistribution studies upon administration of exogenously purified EVs into animal models (Table 1). Recently, imaging of live animals at microscopic resolution (intravital imaging) was used to investigate exosomal cellular trafficking* in vivo* suggesting that EVs take part in the dissemination of cancer cells [66, 67].

### 4.2. *In Vivo* Biodistribution of Exogenously Administered Extracellular Vesicles

The nature and the physiological state of the vesicle-producing cell affect the tropism of produced EVs [58, 109]. Moreover, the characteristics of EVs purified from a defined cellular source cultured* in vitro* may be different from EVs endogenously released from the same source [110]. The lack of standardization in EVs isolation procedures and in the methods for the characterization of the purified fraction hampers direct comparison between different studies. In fact, the isolation method used may substantially affect EVs purity and function and consequently have an impact on the* in vivo* biodistribution. For instance, collection of EVs by ultracentrifugation results in vesicles aggregation [111]. In biodistribution studies, the dosage of administered EVs is mainly assessed by determining the protein content in EVs preparations, which, due to suboptimal isolation protocols, may suffer from protein aggregates contaminations [112]. Furthermore, EVs display an intrinsic broad size distribution and heterogeneity, which may determine differential targeting [37]. Differential posttranslational modifications of EVs membrane proteins are an additional source of variability, which might have a functional role in EVs specific targeting [113]. In addition, purified EVs used for biodistribution studies* in vivo* need to be labeled, and the labeling procedure may modify EVs tropism. Accordingly, the EVs labeling procedure determines the detection method used, with its own advantages and limitations. Further complication in assessing exogenously administered EVs biodistribution is represented by the partial knowledge of the mechanisms of cellular uptake of EVs, recently reviewed by Mulcahy et al. [114]. Nonetheless, from studies summarized in Table 1, some valuable information on pharmacodynamics and biodistribution of administered EVs can be obtained.

Due to their presence in most of biological fluids, it was supposed that EVs may be quite stable in circulation. Unexpectedly, dynamic distribution studies have demonstrated that blood levels of EVs decreased by more than a half from 30 to 60 minutes upon intravenous administration [64]. Pharmacokinetics studies performed by Takahashi et al. suggest a rapid clearance of systemically administered EVs, with half-life of few minutes and complete disappearance from circulation within 4 hours after injection [62]. These results are in accordance with studies performed on systemic administration of liposomes of similar size and charge [110]. Exogenously administered EVs are rapidly cleared predominantly by the macrophages of the mononuclear phagocyte system [62, 63]. Accordingly, EVs clearance is significantly reduced in macrophage depleted animals, compared to animal not subjected to macrophage depletion treatment [63]. In particular, exogenously administered EVs accumulate mainly in liver, spleen, and lungs, organs rich in macrophages (Table 1). Interaction between macrophages and EVs may be mediated by specific phosphatidylserine recognition on the outer portion of the membrane [114, 115]. In the liver, in addition to a predominant clearance by macrophages (Kupffer cells), also direct EVs uptake by hepatocytes has been suggested [63]. Presence of high amounts of systemically delivered EVs into the spleen was attributed to circulating lymphocytes and macrophages, which bind EVs in the blood and then migrate to the spleen [63]. It was observed that EVs are retained in the lungs longer than in other organs, being detectable approximately 4 hours after intravenous delivery [62]. In some experimental conditions, EVs accumulation in the lungs observed after systemic delivery was due to aggregation subsequent to EVs labeling [54]. Exogenously administered EVs may also be internalized by kidney cells and released into the urine [59]. Biodistribution of systemically administered EVs is a dynamic process: a rapid phase of distribution in liver, spleen, and lungs within approximately 30 min upon administration is followed by an elimination phase via hepatic and renal processing, removing EVs in 1 to 6 hours after administration [64, 67].

The route of administration determines EVs biodistribution [58]. For instance, administration into the footpad resulted in EVs localization into lymph nodes [56]; intranasal administration delivered EVs to the brain, across the blood brain barrier [70], opening exciting opportunities on the exploitation of EVs as drug delivery system to the brain [116]; periocular injection of EVs reached the neurosensory retina [117]. Furthermore, it is likely that clearance and organ uptake of EVs may be different in healthy recipients compared to subject suffering some sort of disease or trauma, even if more detailed comparative studies addressing this issue are needed [21].

## 5. Targeting Extracellular Vesicles Delivery

**Figure 2:**
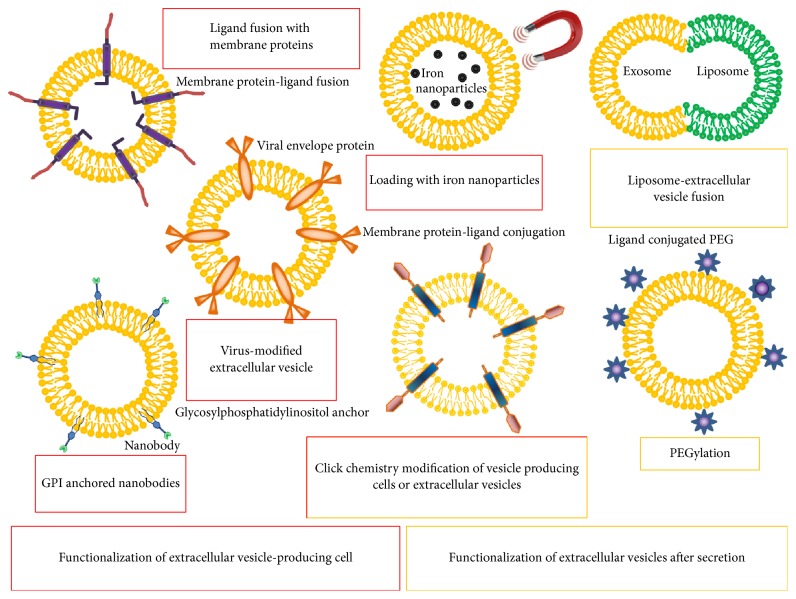
Schematic representation of different methods to promote tissue- or cell-type-specific targeting of extracellular vesicles (EVs). EVs can be targeted to particular cellular receptor either by modifications of EVs-producing cells (red squares) or modification of EVs after secretion (yellow squares). In the first case, EVs-producing cells can be modified: expressing ligands, peptides, or viral-derived envelop proteins in the outer portion of a transmembrane protein; loading cells with iron oxide particles to allow for magnetic targeting. Alternatively, secreted EVs can be modified linking cell-specific peptides to the EVs surface via association with polyethylene glycol (PEG) polymer chains or by EVs-liposome fusion. Click chemistry can be used to modify both EVs-producing cells and purified EVs.

*In vivo* tracking studies have pointed out that, upon systemic delivery, EVs are sequestered within a few minutes by circulating macrophages in the liver, spleen, and lungs [21]. Hence, to achieve a longer half-life of circulating EVs it might be necessary to modify EVs in order to escape macrophage recognition. On the other hand, receptors and ligands exposed on the external part of the lipid bilayer of the membrane play key roles in target cell recognition and EVs uptake [114], although the exact mechanism of specific recipient cell selection has not been fully elucidated [118]. Therefore, detargeting from macrophages or targeting of EVs to specific tissues may enhance their therapeutic efficacy and reduce possible off-target effects. In order to achieve targeted delivery different strategies to modify EVs' natural tropism have been developed (Table 2) [119–121]. Some approaches require the functionalization of the cellular source to generate “tailored” EVs (Figure 2). For instance, targeting restricted cellular receptors can be achieved by genetic modification of the EVs-producing cells, in order to express specific ligands or peptides in the outer portion of a transmembrane protein, such as lactadherin, lysosome-associated membrane protein-2b (LAMP-2b), and platelet-derived growth factor receptor (PDGFR) (Table 2). Using these approaches EVs have been directed to clinical relevant targets such as EGFR-expressing tumors [60], antigen presenting cells [68], and brain [70]. Interestingly, fusion of membrane proteins with specific viral proteins can direct EVs towards specific target cells. Accordingly, Koppers-Lalic and collaborators suggested producing EVs with modified tropism by genetic modification of EVs-secreting cells in order to overexpress viral-derived envelop proteins, taking advantage of viral proteins specific binding to target cell receptors [78]. Albeit effective, it should be considered that such targeting strategies may compromise the function of the EVs, and consequently their therapeutic efficacy, or promote their aggregation [21].

Approaches requiring genetic modification of EVs-secreting cells are cumbersome and time-consuming. In addition, some peptides fused to EVs transmembrane proteins are not effectively exposed or adequately stable to provide for efficient target recognition [122]. Moreover, in some EVs-producing cells, especially primary cells, it might be difficult to achieve a satisfactory level of transgene expression, using both viral and nonviral methods of transduction. To avoid genetic manipulation, Silva et al. loaded the EVs-secreting cells with iron oxide particles to produce EVs-containing magnetic nanoparticles suitable for magnetic targeting [75]. Alternatively, a series of approaches aiming at modifying the EVs after secretion, without the need of manipulating the EVs-producing cells, have been recently pursued (Figure 2). For instance, it is possible to link cell-specific peptides to the EVs surface via association with polyethylene glycol (PEG) polymer chains [77]. The resulting PEGylated EVs are coated with the desired ligand, allowing for specific targeting. PEGylation has the advantage of reducing EVs recognition by the mononuclear phagocytic system. A limitation to the clinical translation of the use of PEGylated EVs for therapeutic purposes is represented by the fact that approximately 25% of healthy subjects are positive to anti-PEG neutralizing antibodies, due to exposure to PEG contained in cosmetic products [123]. Recent studies have provided evidences that click chemistry can be efficiently used to modify EVs-producing cells [81] or purified EVs [82] in order to generate “tailored” vesicles.

Altogether these reports established the possibility to manipulate EV tropism, fostering future studies, in both academia and the pharmaceutical industry, to actively pursue the development of an efficient system with improved target specificity suitable for safe clinical translation.

## 6. Conclusive Remarks

In recent years, stem/stromal mesenchymal cells-derived extracellular vesicles, in particular exosomes, have gained increasing interest and their potential use in regenerative therapies has greatly expanded. Addressing both technical and regulatory issues to bring EVs-based therapies from bench to bedside is an ongoing process. Nonetheless, the exact mechanism of* in vivo* action of exogenously administered EVs, their biodistribution, pharmacokinetics, and possibility of targeted delivery are not fully elucidated. Imaging techniques may help in filling this gap of knowledge and further promoting clinical translation of EVs-based regenerative therapy.

## Figures and Tables

**Table 1:** Imaging studies investigating extracellular vesicles (EVs) biodistribution *in vivo*.

Imaging technique	EVs labeling	EVs source	Administration route	Biodistribution	Ref
PET	^68^Ga and ^64^Cu	Breast cancer cells (4T1)	Tail vein and foot pad	Lung, liver, spleen, lymph nodes	[52]
SPECT/CT	^99m^Tc	Erythrocytes	Tail vein	Liver and spleen	[53]
	^99m^Tc-HMPAO	Macrophages	Tail vein	Liver and spleen	[54]
	^125^I	Melanoma cells (B16BL6)	Intravenous injection	Liver, spleen, lungs	[55]
MRI	Paramagnetic cation probes	Melanoma cells (B16-F10)	Food pad	Lymph node	[56]
	Infrared dye	Mouse lymphoma cell line (EL-4)	Intraperitoneal	Kidney, liver, spleen, lungs	[57]
	Near-infrared dye; GFP labeling	Dendritic cells, MSCs from bone marrow	Tail vein, intraperitoneal, subcutaneous	Liver, spleen, gastrointestinal tract, lungs	[58]
	Near-infrared dye	MSCs	Intravenous injection	Kidney in acute kidney injured mice	[59]
	PKH67 dye	Embryonic kidney cells (HEK293T)	Intravenous injection	Tumor targeting	[60]
Optical imaging					
	Fluorescent dye and ^111^In	Breast cancer cells (4T1)	Tail vein	Liver and spleen	[61]

	gLuc-lactadherin	Melanoma cells (B16BL6)	Tail vein	Liver and lungs	[62]
	gLuc-lactadherin and PKH67 dye	Melanoma cells (B16BL6)	Tail vein	Macrophages in liver and spleen; endothelial cells in lungs	[63]
	gLuc-B and streptavidin-Alexa680	Embryonic kidney cells (HEK293T)	Tail vein	Spleen, liver, lungs, kidney	[64]
Optical imaging and radiolabelling	GFP-tagged CD63	Orthotopically transplanted breast cancer cells	—	Tumor	[65]
Intra vital imaging	Cre-GFP-RFP	Orthotopically transplanted MDA-MB-231	—	Tumor	[66]
	PalmGFP, PalmtdTomato	Mouse lymphoma cell line (EL-4)	Intratumor injection	Tumor	[67]

HMPAO: hexamethylpropyleneamine oxime. gLuc-lactadherin: Gaussia luciferase and a truncated lactadherin reporter. gLuc-B: fusion between a membrane-bound variant of the Gluc reporter and a biotin acceptor peptide.

**Table 2:** Extracellular vesicles targeting studies.

Target cells	Ligand	Receptor	Main reference
APCs	Lactadherin-fusion	Antigen targeting	[68, 69]
Neurons	RVG-Lamp2b fusion	Acetylcholine receptor	[70–72]
B cells	EBV glycoprotein 350	CD19	[73]
Breast cancer	PDGFR-GE11 peptide fusion	EGFR	[60]
	RGD-	*α*v*β*3 integrins	[74]
Cancer cells	Iron oxide nanoparticles	Magnetic targeting	[75]
Carcinoma cells	Nanobodies anti-EGFR fused to GPI anchors	EGFR	[76]
	Nanobodies anti-EGFR conjugated with PEG		[77]
Different targets	Viral envelope proteins	Dependent on the type of the virus	[78]
	Exosome fusion with liposomes	Dependent on the type of the hybrid exosome	[79, 80]
	Click chemistry modification	Dependent on the type of the functionalization	[81, 82]
